# Young carer knowledge scale: Development, validation, and implications for support

**DOI:** 10.1002/pcn5.70194

**Published:** 2025-08-27

**Authors:** Masateru Matsushita, Wakana Kurosaka, Asuka Koyama

**Affiliations:** ^1^ Department of Psychology, Faculty of Psychology Konan Women's University Kobe Japan; ^2^ Department of Psychiatry Kinki Central Hospital of the Mutual Aid Association of Public School Teachers Itami Japan; ^3^ Department of Clinical Psychology Naruto University of Education Naruto Japan; ^4^ Faculty of Social Welfare Kumamoto Gakuen University Kumamoto Japan

**Keywords:** public awareness, psychosocial burden, reliability and validation, support policies, young caregivers

## Abstract

**Aim:**

“Young carers (YCs)” are children under the age of 18 who undertake adult caregiving roles, including household chores, family care, nursing, and emotional support. Despite the growing attention to YCs in Japan, public understanding remains limited. This study aims to develop a scale to measure YCs' knowledge and examine its reliability and validity.

**Methods:**

A web‐based survey was conducted in October 2023 with 300 participants aged 22–70 years. Participants were divided into three groups based on their subjective awareness of YCs: unaware, aware but unfamiliar, and aware and familiar. After excluding dishonest responses, data from 218 individuals (mean age = 53.2 ± 10.5 years) were analyzed. A 57‐item questionnaire was developed, and exploratory factor analysis (principal factor method, Promax rotation) was conducted. Reliability was assessed using Cronbach's *α*, and discriminant validity was examined through multivariate analysis of covariance (MANCOVA), controlling for gender, marital status, and parental status.

**Results:**

Factor analysis identified 29 items across three factors: excessive family roles, care‐related daily life challenges, and hidden impacts of caring. The scale showed high internal consistency (*α* = 0.91–0.97). MANCOVA revealed significant differences in the knowledge scores according to subjective awareness (Wilks' Λ = 0.71, *F*(6, 420) = 9.22, *p* < 0.001), supporting discriminant validity. Greater awareness of YCs was associated with greater knowledge of YCs' circumstances.

**Conclusion:**

These findings suggest that this scale may help assess the public understanding of YCs. Future studies should investigate variations in YCs' knowledge across different occupations, beliefs, and cultural contexts.

## Graphical abstract

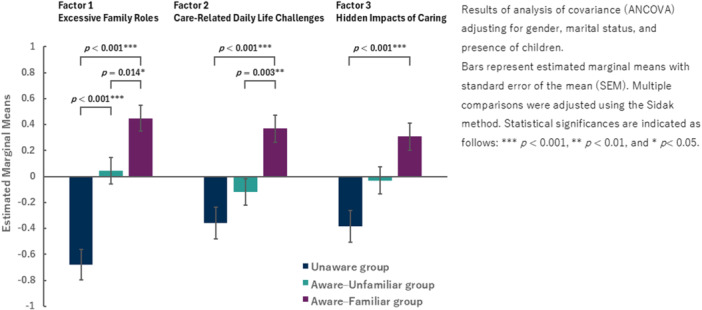

## INTRODUCTION

When a family member requires support—for instance, owing to mental illness, disability, or limited proficiency in a foreign language—children may consistently assume adult caregiving roles, including daily household chores, personal care tasks, and providing emotional support.^1^ Children in these situations are referred to as “young carers (YCs),” and their experiences have recently become a focus of research in Japan.^2^ With the rise of nuclear family structures and the promotion of home‐based caregiving, it has been suggested that children and younger generations are increasingly likely to assume caregiving responsibilities.^3^

The roles fulfilled by YCs are diverse, encompassing physical care, daily assistance, household maintenance, shopping, processing bills, interpreting for family members who do not speak Japanese as their first language, and caring for siblings. Despite these substantial and ongoing responsibilities, YCs may not perceive their situation as problematic. For example, in a survey conducted among high school students, 26.8% of those who did not consider themselves YCs acknowledged providing care for needy family members.^2^ This finding indicates that there are quite a few cases where individuals are YCs but are unaware of their caregiving roles. Furthermore, another survey suggested that a certain number of elementary school students who reported caring for their family members had their health and school life negatively impacted.^4^ Notably, the findings confirm that the more time spent on caregiving, the more pronounced the impact on daily life and academic performance, and the greater the sense of burden experienced by the individual.^4^ It is generally believed that the younger the YCs are, the more difficult it becomes for them to understand their caregiving role. They often perceive family caregiving as natural and, consequently, are less likely to seek advice or support from others.

Furthermore, individuals often struggle to recognize themselves as YCs, while those around them may overlook their situation, contributing to inadequate support. Children who take on roles within the household are often perceived as “well‐behaved” or “helpful,” which makes the severity of their caregiving responsibilities less visible. This perception tends to obscure their roles, limiting public understanding and awareness of YCs in Japan. According to a nationwide survey, only 29.8% of respondents had heard the term “young carer” and understood its meaning.^4^ Additionally, when asked how to assist children who may be YCs, the most common response was “I do not know” (39.9%). The survey also indicated a trend where lower levels of awareness corresponded with an increased proportion of “do nothing” or “uncertain” responses.^4^ Therefore, raising public awareness and promoting education about YCs has become an urgent social challenge.

The Young Carers Scale Japanese version (YCS‐J) is an evaluation tool for assessing YCs in Japan.^5^ The YCS‐J assesses caregiving activities and identifies whether respondents are YCs. However, no evaluation tools have been developed in Japan to assess how YCs are perceived by their surroundings. Prejudice against YCs and a limited understanding of their caregiving roles may hinder their access to adequate support, emphasizing the importance of public education and awareness. Therefore, developing scales that enable a more objective evaluation of knowledge about YCs is essential for promoting appropriate support for YCs and fostering deeper societal understanding.

This study aimed to develop and validate a scale for objectively evaluating the knowledge of YCs, focusing on its reliability and validity.

## METHODS

### Participants and procedure

The survey was conducted in October 2023 using a web‐based survey panel with 300 men and women aged 22–70 years residing throughout Japan. Data were collected regarding the participants' demographics (age, sex, marital status, and parental status), subjective awareness of YCs, and knowledge of YCs.

Participants were divided into three groups based on their subjective awareness of YCs: an unaware group (those who had never heard of YCs, *n* = 100), an aware‐unfamiliar group (those who had heard of YCs but were not familiar with them, *n* = 100), and an aware‐familiar group (those who were familiar with the concept of YCs, *n* = 100). Data were gathered from 300 participants, consisting of 100 individuals each from the unaware, aware‐unfamiliar, and aware‐familiar groups.

The collected data excluded responses suspected of dishonesty and cases where participants failed to appropriately answer two items on the Directed Questions Scale (DSQ).^6^ Consequently, the final analysis included data from 218 individuals (65 women, representing 29.8%, with a mean age of 53.2 ± 10.5 years), yielding a valid response rate of 72.7%.

The study was approved by the Ethics Committee of Konan Women's University (Approval No. 2023035). After receiving a full explanation, the participants provided informed consent by completing the survey.

### Measurement

#### Subjective awareness of YCs

Subjective awareness of YCs was assessed with the question, “Have you ever heard the term ‘young carers’?” Participants were given three response options: “1. Never heard of it,” “2. Heard of it but not familiar with the details,” and “3. Heard of it and am familiar with the concept.” Participants who chose the first option were defined as the unaware group. Those who selected the second option were defined as the aware‐unfamiliar group, and those who chose the third option were defined as the aware‐familiar group.

#### YC knowledge scale

The development of a questionnaire to measure knowledge about YCs was based on the findings of Untas et al. and other reports on the factual investigation of YCs.^2^, ^4^, ^7^, ^8^, ^9^, ^10^, ^11^, ^12^ The initial questionnaire comprised 57 items designed to assess respondents' knowledge of YCs. It included the following sections: the characteristics of YCs (Q1: 23 items), the causes and roles of becoming a YC (Q2: 11 items), the school life of YCs (Q3: 9 items), and other matters related to YCs (Q4: 14 items). Respondents rated the extent of their agreement on a four‐point Likert scale (“agree (4),” “somewhat agree (3),” “somewhat disagree (2),” and “disagree (1)”). This survey included reverse‐scored items that were adjusted by reversing their scores to ensure consistency in direction before analysis. Higher scores reflected a greater level of knowledge of YCs.

#### Detection of dishonest responses

Two DQS items were incorporated to ensure participants' attentiveness.^6^ These items were designed to prompt participants to select a specific response, detecting carelessness or dishonesty. For example, one item explicitly instructed the participants to choose “somewhat disagree” as their answer.

### Statistical analysis

Statistical analyses were conducted using IBM SPSS Statistics 23.0 for Windows (IBM Corp., Armonk, NY), with a significance level of *p* < 0.05.

Group comparisons were conducted using analysis of variance (ANOVA), *χ*² tests, *z*‐tests, and multivariate analysis of covariance (MANCOVA). Analysis of covariance (ANCOVA) was used for additional comparisons.

For factor analysis, factors were extracted using the principal factor method, followed by Promax rotation. The number of factors was determined based on the rate of eigenvalue reduction, and items with factor loadings of at least 0.4 were retained. Items with high loadings for multiple factors or insufficient loadings for any other factor were excluded. Factor scores were calculated using regression analysis.

Kaiser–Meyer–Olkin (KMO) and Bartlett's test of sphericity were conducted to assess the suitability of the factor analysis. The KMO values ranged from 0 to 1, with values above 0.5 indicating that the data were suitable for factor analysis. Bartlett's test of sphericity, with a *p*‐value below 0.05, showed significant correlations among the variables, supporting the validity of the factor analysis.

Cronbach's *α* was calculated to ensure the internal consistency of each factor. A MANCOVA was conducted to examine how subjective awareness levels of the YCs affect the factor scores of the YCs' knowledge components. Subjective awareness levels served as independent variables, and factor scores as dependent variables. Sex, marital status, and presence of children were included as covariates. Multiple comparisons were conducted using ancova with Bonferroni correction and paired tests with a Sidak adjustment.

## RESULTS

Table 1 presents the demographic characteristics of the participants. Comparisons revealed that subjective awareness of YCs was significantly different across sex, marital status, and parental status, but not age. The findings indicated that women, especially those who were married and had children, tended to show a higher subjective awareness of YCs.

**Table 1:** Characteristics of study participants.

	Unaware Group A (*n* = 59)		Aware‐unfamiliar Group B (*n* = 77)		Aware‐familiar Group C (*n* = 82)		*F*/*χ* ^2^	*p*	*z*‐test
Age, mean (SD)	54.9	9.75	53.0	10.33	52.2	11.25	1.182	0.309n.s.	
Sex, *n* (%)									
Men	49	83.1%	56	72.7%	48	58.5%	10.222	0.006**	Group A ≠ Group C
Women	10	16.9%	21	27.3%	34	41.5%			
Marital status, *n* (%)									
Married	21	35.6%	41	53.2%	50	61.0%	9.016	0.011*	Group A ≠ Group C
Unmarried	38	64.4%	36	46.8%	32	39.0%			
Parental status, *n* (%)									
With children	21	35.6%	36	46.8%	47	57.3%	6.534	0.038*	Group A ≠ Group C
Without children	38	64.4%	41	53.2%	35	42.7%			

* *p* < 0.05

** *p* < 0.01.

Floor effects occur when the value calculated by subtracting one standard deviation from the mean falls below the lower limit of the score range. In contrast, ceiling effects occur when one standard deviation added to the mean exceeds the upper limit. No ceiling effects were observed in this analysis. However, 19 items that exhibited floor effects were excluded from the subsequent factor analysis to avoid potential measurement bias. An exploratory factor analysis was then conducted on the remaining 38 items related to YCs using the principal factor method and Promax rotation. The number of factors was determined to be three based on the eigenvalue decay. During the analysis, items with high loadings on multiple factors or low loadings on a single factor were excluded, resulting in the removal of nine items. The final structure consisted of three factors and 29 items. The KMO measure of sampling adequacy was 0.96, indicating that the data were suitable for factor analysis. Bartlett's test of sphericity also confirmed the adequacy of the data for factor analysis (*χ*²(1431) = 11,980.4, *p* < 0.001).

Table 2 presents the results of the factor analysis. The first factor, labeled “*excessive family roles*,” comprised 14 items that reflect the diverse and ongoing caregiving responsibilities undertaken by YCs. These items include “managing the illness of a family member with disabilities or chronic conditions,” “providing routine medical care,” “caring for family members with chronic illnesses,” “monitoring and supporting family members,” “looking after siblings,” “contributing to household finances through work,” “assisting with family transportation,” and “handling administrative procedures for government agencies.” These items highlighted the characteristics of YCs compelled to assume adult household roles. Cronbach's *α* for this factor was 0.97, indicating high internal consistency.

**Table 2:** Factor loadings for item assessing knowledge about young carers.

Items		Factor 1	Factor 2	Factor 3	Communalities
		EFR	CRDLC	HIC	
Q1	Some YCs assist family members with disabilities or illnesses by managing medical appointments and picking up prescriptions.	**0.97**	−0.04	−0.07	0.83
Q1	Some YCs provide medical care for family members with disabilities or illnesses, including managing medication or performing suctioning procedures.	**0.97**	−0.08	0.00	0.85
Q1	Some YCs care for family members with chronic illnesses, for instance, those with cancer, intractable diseases, or mental health conditions.	**0.94**	0.06	−0.08	0.86
Q1	Some YCs monitor and offer verbal reassurance to family members who require constant attention.	**0.93**	0.08	−0.12	0.82
Q1	Some YCs care for and watch over siblings with disabilities or illnesses.	**0.92**	0.00	0.00	0.85
Q1	Some YCs work to support the family and assist relatives with disabilities or illnesses.	**0.90**	−0.04	0.06	0.84
Q1	YCs may help family members with transportation or carrying luggage to support their mobility.	**0.86**	0.05	0.04	0.83
Q1	YCs may struggle to find time for studies, limiting their ability to plan for higher education.	**0.73**	0.29	−0.11	0.71
Q1	YCs may complete procedures at government offices, such as paying bills, to assist their families.	**0.72**	−0.14	−0.01	0.41
Q1	Some YCs talk with their siblings' teachers about school matters on behalf of their parents.	**0.69**	0.00	0.06	0.53
Q1	Some YCs interpret for family members with language barriers or disabilities.	**0.67**	−0.23	0.21	0.49
Q2	Because their parents have disabilities or mental health conditions, they have taken on the role of YCs.	**0.63**	0.12	0.12	0.64
Q1	Some YCs support family members emotionally by listening and offering encouragement.	**0.63**	−0.11	0.14	0.44
Q2	Given that no one else is available to provide care, YCs have assumed caregiving responsibilities.	**0.57**	0.22	0.08	0.61
Q3	Some YCs are late to school due to caregiving responsibilities.	−0.09	**0.98**	−0.10	0.76
Q3	Some YCs lack sufficient time for their studies because of family and caregiving duties.	0.04	**0.82**	0.02	0.74
Q3	Some YCs struggle to complete homework owing to family duties.	−0.11	**0.82**	0.12	0.70
Q3	Some YCs struggle to maintain friendships because of home responsibilities.	0.08	**0.79**	0.02	0.73
Q3	Caregiving and living situations can make it difficult to maintain proper hygiene.	−0.07	**0.74**	0.10	0.60
Q3	Family circumstances may sometimes require leaving school early.	−0.01	**0.69**	0.05	0.52
Q4	YCs may worry about the possibility of their family members being placed in facilities and separated from them.	−0.11	0.07	**0.80**	0.62
Q4	Through caregiving, YCs may develop a more mature mindset or personality than their peers.	0.02	0.01	**0.76**	0.61
Q4	YCs' caregiving responsibilities often include household chores and emotional support for their family.	0.05	−0.02	**0.73**	0.56
Q4	YCs sharing their experiences may sometimes make their parents feel embarrassed.	−0.04	0.09	**0.70**	0.55
Q4	When YCs share their experiences, it may sometimes lead to their parents being criticized by others.	−0.05	0.13	**0.69**	0.56
Q4	Parents of YCs may sometimes feel guilty for depending on their children for caregiving.	0.15	−0.05	**0.61**	0.47
Q4	Sometimes, the care provided by YCs is mistaken for ordinary family help, instead of the essential support it truly is.	0.12	−0.03	**0.60**	0.43
Q4	Some YCs manage caregiving responsibilities while living with developmental disabilities themselves.	0.22	0.08	**0.55**	0.59
Q4	As YCs get older, their caregiving roles tend to increase.	0.21	0.24	**0.42**	0.58
	Eigenvalue	14.84	2.77	1.13	
	Proportion of variance explained	51.17	9.56	3.88	
	Cumulative variance explained	51.17	60.73	64.61	

*Note*: Factor loadings obtained using principal axis factoring with promax rotation. The bold numbers indicate a factor loading of 0.4 or higher.

Abbreviations: CRDLC, care‐related daily life challenges; EFR, excessive family roles; HIC, hidden impacts of caring; YCs, young carers.

The second factor, labeled “*care‐related daily life challenges*,” comprised six items that reflect YCs' challenges in school due to their caregiving responsibilities at home. These items include “a tendency to be frequently late or leave early,” “difficulty securing time to study,” “challenges in completing homework,” “negative impacts on relationships with friends and classmates,” and “inability to maintain personal grooming and hygiene.” These factors underscore the educational and social disadvantages YCs experience due to their caregiving roles. Cronbach's *α* for this factor was 0.92, indicating high internal consistency.

The third factor, labeled “*hidden impacts of caring*,” consisted of nine items that reflect the psychosocial challenges faced by YCs, which are not readily visible but are of significant importance. These items include “concerns about family members being placed in care facilities by others,” “impacts on personal development,” “fear that sharing their experiences may lead to criticism or stigma for their parents,” “parents feeling a sense of guilt,” “care activities being perceived as mere ‘helping out’,” and “the presence of developmental disabilities in the carer.” These items underscore the hidden emotional and social burdens YCs carry as part of their caregiving roles. Cronbach's *α* for this factor was 0.91, indicating high internal consistency.

The communality values for each variable ranged from 0.41 to 0.86, indicating that the factors adequately explained the variability in the variables.

Table 3 presents each item's means, standard deviations, and standard errors. The mean scores for the items in Factor 2 (*care‐related daily life challenges*) and Factor 3 (*hidden impacts of caring*) tended to be lower than those in Factor 1.

**Table 3:** Descriptive statistics of each item.

Factors	Items		Mean	SD	SE
1. EFR	Q1	Some YCs assist family members with disabilities or illnesses by managing medical appointments and picking up prescriptions.	3.04	0.874	0.059
1. EFR	Q1	Some YCs provide medical care for family members with disabilities or illnesses, including managing medication or performing suctioning procedures.	3.07	0.875	0.059
1. EFR	Q1	Some YCs care for family members with chronic illnesses, for instance, those with cancer, intractable diseases, or mental health conditions.	3.06	0.916	0.062
1. EFR	Q1	Some YCs monitor and offer verbal reassurance to family members who require constant attention.	3.07	0.89	0.06
1. EFR	Q1	Some YCs care for and watch over siblings with disabilities or illnesses.	3.11	0.854	0.058
1. EFR	Q1	Some YCs work to support the family and assist relatives with disabilities or illnesses.	3.02	0.872	0.059
1. EFR	Q1	YCs may help family members with transportation or carrying luggage to support their mobility.	3.11	0.859	0.058
1. EFR	Q1	YCs may struggle to find time for studies, limiting their ability to plan for higher education.	3.10	0.863	0.058
1. EFR	Q1	YCs may complete procedures at government offices, such as paying bills, to assist their families.	2.68	0.91	0.062
1. EFR	Q1	Some YCs talk with their siblings' teachers about school matters on behalf of their parents.	2.67	0.93	0.063
1. EFR	Q1	Some YCs interpret for family members with language barriers or disabilities.	2.68	0.878	0.059
1. EFR	Q2	Because their parents have disabilities or mental health conditions, they have taken on the role of YCs.	3.12	0.858	0.058
1. EFR	Q1	Some YCs support family members emotionally by listening and offering encouragement.	2.70	0.931	0.063
1. EFR	Q2	Given that no one else is available to provide care, YCs have assumed caregiving responsibilities.	3.16	0.839	0.057
1. EFR	Q3	Some YCs are late to school due to caregiving responsibilities.	2.64	0.837	0.057
2. CRDLC	Q3	Some YCs lack sufficient time for their studies because of family and caregiving duties.	2.81	0.803	0.054
2. CRDLC	Q3	Some YCs struggle to complete homework owing to family duties.	2.65	0.802	0.054
2. CRDLC	Q3	Some YCs struggle to maintain friendships because of home responsibilities.	2.81	0.814	0.055
2. CRDLC	Q3	Caregiving and living situations can make it difficult to maintain proper hygiene.	2.58	0.818	0.055
2. CRDLC	Q3	Family circumstances may sometimes require leaving school early.	2.65	0.808	0.055
3. HIC	Q4	YCs may worry about the possibility of their family members being placed in facilities and separated from them.	2.57	0.766	0.052
3. HIC	Q4	Through caregiving, YCs may develop a more mature mindset or personality than their peers.	2.78	0.797	0.054
3. HIC	Q4	YCs' caregiving responsibilities often include household chores and emotional support for their family.	2.71	0.807	0.055
3. HIC	Q4	YCs sharing their experiences may sometimes make their parents feel embarrassed.	2.63	0.805	0.055
3. HIC	Q4	When YCs share their experiences, it may sometimes lead to their parents being criticized by others.	2.72	0.802	0.054
3. HIC	Q4	Parents of YCs may sometimes feel guilty for depending on their children for caregiving.	2.77	0.745	0.05
3. HIC	Q4	Sometimes, the care provided by YCs is mistaken for ordinary family help, instead of the essential support it truly is.	2.72	0.809	0.055
3. HIC	Q4	Some YCs manage caregiving responsibilities while living with developmental disabilities themselves.	2.75	0.764	0.052
3. HIC	Q4	As YCs get older, their caregiving roles tend to increase.	2.92	0.751	0.051

Abbreviations: CRDLC, care‐related daily life challenges; EFR, excessive family roles; HIC, hidden impacts of caring; SD, standard deviation; SE, standard error; YCs, young carers.

To assess the validity of knowledge about YCs, differences in the three factor scores based on awareness of YCs were examined using MANCOVA, with sex, marital status, and the presence of children included as covariates. The results of the MANCOVA indicated a significant main effect between groups (Wilks' Λ = 0.71, *F*(6, 420) = 9.22, *p* < 0.001, *η*² = 0.116). This suggests that knowledge about YCs was appropriately differentiated, as differences between the groups remained even after controlling for the covariates. None of the covariates significantly affected the dependent variables.

ancova comparisons for each dependent variable revealed statistically significant differences between groups based on subjective awareness for the first factor score (*F*(2, 212) = 26.15, *p* < 0.001, *η*² = 0.198), the second factor score (*F*(2, 212) = 10.96, *p* < 0.001, *η*² = 0.094), and the third‐factor score (*F*(2, 212) = 9.06, *p* < 0.001, *η*² = 0.079). The covariates did not significantly influence any of the three dependent variables.

Multiple comparisons, adjusted using the Sidak correction, indicated significant differences in factor scores between the groups (Table 4).

**Table 4:** Multivariate Analysis of Covariance (MANCOVA) comparing factor scores based on awareness of young carers.

	Unaware group		Aware‐unfamiliar group		Aware‐familiar group		ancova		MANCOVA		
	EMM	SD	EMM	SD	EMM	SD	*F*_2, 212_	Partial *η* ^2^	Wilks' Λ	*F*_6, 420_	Partial *η* ^2^
F1. Excessive family roles	−0.68	0.117	0.04	0.100	0.45	0.099	25.15***	0.198	0.71	9.22***	0.116
F2. Care‐related daily life challenges	−0.36	0.121	−0.12	0.104	0.37	0.103	10.96***	0.094			
F3. Hidden impacts of caring	−0.39	0.122	−0.03	0.104	0.31	0.103	9.06***	0.079			

*Note*: Sex, marital status, and parental status are included as covariates.

Abbreviations: ANCOVA, analysis of covariance; EMM, estimated marginal means; MANCOVA, multivariate analysis of covariance; SD, standard deviation.

*** *p* < 0.001.

In the scores for the first factor, “*excessive family roles*,” the aware‐familiar group scored significantly higher than the unaware group (mean difference [MD] = 1.129, *p* < 0.001, 95% confidence interval [CI] [0.752, 1.506]). Additionally, the aware‐familiar group scored significantly higher than the aware‐unfamiliar group (MD = 0.405, *p* = 0.014, 95% CI [0.065, 0.744]). Furthermore, the aware‐unfamiliar group scored significantly higher than the unaware group (MD = 0.724, *p* < 0.001, 95% CI = [0.354, 1.095]).

In the scores for the second factor, “*care‐related daily life challenges*,” the aware‐familiar group scored significantly higher than the unaware group (MD = 0.725, *p* < 0.001, 95% CI [0.335, 1.116]). Furthermore, the aware‐familiar group scored significantly higher than the aware‐unfamiliar group (MD = 0.487, *p* = 0.003, 95% CI [0.135, 0.839]).

**Figure 1:**
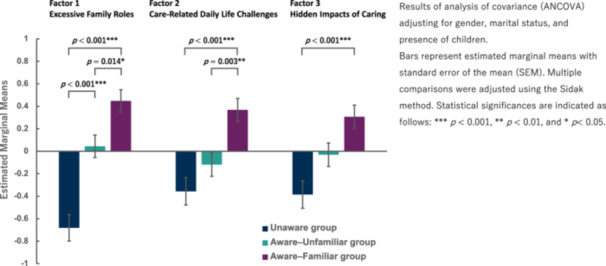
Comparison of factor scores based on awareness of young carers.

In the scores for the third factor, “*hidden impacts of caring*,” the aware‐familiar group scored significantly higher than the unaware group (MD = 0.692, *p* < 0.001, 95% CI [0.299, 1.085]). These results support the validity of the YC knowledge scale in differentiating the levels of knowledge of YCs among groups with varying degrees of awareness (Figure 1).

## DISCUSSION

This study aimed to develop and validate a new scale for objectively measuring the knowledge of YCs. Data obtained from an online survey targeting the general population were subjected to exploratory factor analysis, resulting in the extraction of a structure comprising three factors: “*excessive family roles*,” “*care‐related daily life challenges*,” and “*hidden impacts of caring*,” with 29 items. Furthermore, significant differences in factor scores were observed based on the level of subjective awareness of YCs, suggesting that this scale appropriately reflects the degree of knowledge regarding YCs.

This study revealed a lack of knowledge about YCs in the general population. In particular, knowledge related to the “*care‐related daily life challenges*” and “*hidden impacts of caring*” was insufficient. These areas should be emphasized in awareness‐raising initiatives to ensure a more comprehensive understanding of the challenges faced by YCs. As noted by Becker, YCs are considered "hidden populations" in many countries, and the present findings are consistent with this observation.^13^ Similarly, previous studies, such as those by Untas et al. and domestic research reports, have shown that insufficient awareness often leads to underestimating YCs' circumstances and challenges.^4^, ^7^ In line with these findings, the present study found that groups with lower awareness levels demonstrated inadequate understanding of “*excessive family roles*” and the “*care‐related daily life challenges*.” Additionally, as highlighted by Nap et al., YCs often fail to recognize their caregiving roles, which limits their access to support.^14^ Warren also noted that YCs' experiences can profoundly impact academic achievement and future planning.^15^ These observations underscore the importance of raising awareness and strengthening support measures within educational settings. This study reinforces previous findings by suggesting that limited knowledge may hinder the recognition of YCs.

Notably, significant differences in the knowledge scores for the “*hidden impacts of caring*” factor were also observed according to levels of awareness, representing a novel contribution that suggests variations in understanding exist among the general public and highlights the importance of addressing psychosocial challenges and physical caregiving burdens in awareness‐raising efforts for supporting YCs.

Acknowledging the positive aspects of caregiving motivation, such as a “sense of caring for their family” and a “desire to be appreciated for their efforts,” is crucial. Aoki emphasized that positive recognition from others can enhance YCs' self‐efficacy and that caregiving is not necessarily perceived purely as a burden.^3^ Thus, support efforts aimed solely at burden reduction may risk disregarding YCs' perspectives and result in undesired interventions. Appropriate support should involve empathetic listening and flexible, individualized responses. Furthermore, Smyth et al.^16^ pointed out that cultural norms that frame caregiving as a natural family role contribute to the invisibility of YCs' issues. Therefore, support approaches must respect the positive aspects of caregiving experiences while considering cultural contexts and connecting YCs to the necessary assistance without imposing external assumptions.

The findings indicate that individuals with greater awareness of YCs tend to have a better understanding of the multifaceted realities, such as “*excessive family roles*,” “*care‐related daily life challenges*,” and “*hidden impacts of caring*.” This suggests that future awareness campaigns and support policies should promote familiarity with the term “young carers” and deepen the public understanding of YCs' specific living conditions and psychological impacts. Moreover, analyzing score patterns on the scale may offer practical applications for identifying target populations for YC support and exploring effective intervention strategies.

## STRENGTHS AND LIMITATIONS

The strengths of this study include the development of a comprehensive and objective scale for measuring knowledge about YCs, as well as the statistical validation of its reliability and validity. Furthermore, by conducting group comparisons based on awareness levels, the study confirmed the scale's discriminant validity.

However, this study has several limitations. First, subjective awareness of YCs was assessed solely via self‐report questionnaires, which, while insightful, are prone to biases such as social desirability and recall bias. Consequently, subjective awareness in this study may lack accuracy and objectivity. To enhance the scale's validity, future studies should include professionals with practical YC support experience, such as teachers and healthcare workers, and compare their knowledge with that of the general public. Second, this study focused on measuring the general public's knowledge of YCs. Accordingly, the questionnaire did not directly address “barriers to accessing support systems” or “delays in policy responses,” as highlighted in previous research.^17^, ^18^, ^19^ Given that most participants were expected to have limited familiarity with such systems, it was challenging to ensure validity for those items. Therefore, further research involving professionals and individuals with lived experience of YCs is necessary to clarify the relationship between policy awareness and general knowledge. Lastly, since this study employed a cross‐sectional design, it did not assess changes in knowledge over time or the effects of awareness‐raising interventions. Future longitudinal research is needed to examine how targeted interventions addressing specific knowledge gaps (e.g., “*hidden impacts of caring*”) influence knowledge levels and to validate the scale's applicability over time.

## CONCLUSION

This study developed an innovative and objective scale to measure knowledge about YCs. The scale's reliability and validity were confirmed through extensive evaluation, providing a robust tool for advancing research and policy development. The scale is expected to be applied in broader contexts, such as regional and occupational comparisons, the evaluation of awareness intervention effects, and international comparative studies. It is also anticipated that the scale will serve as a foundation for promoting education about YCs among adults and strengthening social support systems, alongside direct assistance for YCs themselves.

## AUTHOR CONTRIBUTIONS

Masateru Matsushita and Wakana Kurosaka wrote the manuscript. Masateru Matsushita and Wakana Kurosaka analyzed the data. Asuka Koyama conceptualized this study. All authors contributed to the manuscript revision and approved the final version.

## CONFLICT OF INTEREST STATEMENT

The authors declare no conflicts of interest.

## ETHICS APPROVAL STATEMENT

This study was conducted in accordance with ethical guidelines and approved by the Ethics Committee of Konan Women's University (Approval No. 2023035).

## PATIENT CONSENT STATEMENT

Written informed consent was not obtained for this study. Participants were provided with a detailed explanation of the study's purpose and procedures. Participants were considered to have given their consent by voluntarily participating after reading the explanation.

## CLINICAL TRIAL REGISTRATION

N/A.

## Data Availability

The datasets generated or analyzed in the current study are available from the corresponding author upon reasonable request. Requests to access the datasets can be directed to Masateru Matsushita.
